# Virtually Possible: Enhancing Quality Control of 3D-Printed Medicines with Machine Vision Trained on Photorealistic Images

**DOI:** 10.3390/pharmaceutics15112630

**Published:** 2023-11-16

**Authors:** Siyuan Sun, Manal E. Alkahtani, Simon Gaisford, Abdul W. Basit, Moe Elbadawi, Mine Orlu

**Affiliations:** 1 UCL School of Pharmacy, University College London, 29-39 Brunswick Square, London WC1N 1AX, UK; siyuan.sun.22@ucl.ac.uk (S.S.); manal.alkahtani.17@ucl.ac.uk (M.E.A.); s.gaisford@ucl.ac.uk (S.G.);; 2 Department of Pharmaceutics, College of Pharmacy, Prince Sattam bin Abdulaziz University, Alkharj 11942, Saudi Arabia; 3 School of Biological and Behavioural Sciences, Queen Mary University of London, Mile End Road, London E1 4DQ, UK

**Keywords:** Industry 5.0, Healthcare 5.0, quality control, computer vision, digital twin, digital manufacturing, virtual technology, additive manufacturing, drug development, personalized formulations, oral drug products

## Abstract

Three-dimensional (3D) printing is an advanced pharmaceutical manufacturing technology, and concerted efforts are underway to establish its applicability to various industries. However, for any technology to achieve widespread adoption, robustness and reliability are critical factors. Machine vision (MV), a subset of artificial intelligence (AI), has emerged as a powerful tool to replace human inspection with unprecedented speed and accuracy. Previous studies have demonstrated the potential of MV in pharmaceutical processes. However, training models using real images proves to be both costly and time consuming. In this study, we present an alternative approach, where synthetic images were used to train models to classify the quality of dosage forms. We generated 200 photorealistic virtual images that replicated 3D-printed dosage forms, where seven machine learning techniques (MLTs) were used to perform image classification. By exploring various MV pipelines, including image resizing and transformation, we achieved remarkable classification accuracies of 80.8%, 74.3%, and 75.5% for capsules, tablets, and films, respectively, for classifying stereolithography (SLA)-printed dosage forms. Additionally, we subjected the MLTs to rigorous stress tests, evaluating their scalability to classify over 3000 images and their ability to handle irrelevant images, where accuracies of 66.5% (capsules), 72.0% (tablets), and 70.9% (films) were obtained. Moreover, model confidence was also measured, and Brier scores ranged from 0.20 to 0.40. Our results demonstrate promising proof of concept that virtual images exhibit great potential for image classification of SLA-printed dosage forms. By using photorealistic virtual images, which are faster and cheaper to generate, we pave the way for accelerated, reliable, and sustainable AI model development to enhance the quality control of 3D-printed medicines.

## 1. Introduction

Three-dimensional (3D) printing, also known as additive manufacturing, has emerged as a revolutionary technology in the pharmaceutical industry. While traditional pharmaceutical manufacturing adopts the paradigm of ‘one size fits all’, where large-scale manufacturing leads to identical dosage forms, the advent of 3D printing enables the production of small batches and on-demand fabrication of more personalized medicines [1,2]. It has several advantages over conventional manufacturing methods, including the ability to fabricate complex geometries with high precision, rapid prototyping, and reduced waste and cost [3,4,5,6,7,8,9]. Three-dimensional printing grants the ability to tailor medicines seamlessly to meet the individual patient’s needs, thereby enhancing the safety and efficacy of therapeutics [10]. The technology has the potential to achieve the on-demand manufacturing of personalized medical products at the point of care (PoC), where hospitals and healthcare providers can have their own 3D printers to produce customized medical devices or drug formulations as needed [11]. However, the PoC manufacturing of personalized medicines using 3D printing technologies has partially been hindered by the challenges associated with its quality control (QC).

QC is a crucial aspect of pharmaceutical manufacturing, ensuring that the product is safe, effective, and its production process is consistent and reproducible [12]. Existing traditional QC methods for large-scale manufacturing follow an end-product testing paradigm, which is usually destructive [13]. Thus, this requires additional dosage forms fabricated for QC purposes, which may not be suitable for the 3D printing of costly drugs [11]. With the transition from batch production to continuous production in the pharmaceutical industry, process analytical technology (PAT) has gained increasing research interest as a quality control method [14]. PAT provides the opportunity to monitor pharmaceutical manufacturing processes by real-time measurements and the control of predefined critical quality attributes and critical process parameters [15]. The widespread adoption of PAT by the pharmaceutical industry has been driven by regulatory guidelines like the FDA Pharmaceutical Quality for the 21st Century Initiative [16]. The application of PAT in pharmaceutical 3D printing aligns with the FDA’s emphasis on QC, traceability, and risk management, which will ensure that the 3D-printed products meet the regulatory standards for safety and efficacy [17]. This necessitates the development of non-destructive PAT, which maintains the integrity of the final product and requires minimal sample preparation. Researchers have explored the use of different PAT tools such as Near-Infrared Spectroscopy (NIR) and Raman Spectroscopy; however, these techniques are hindered by high costs and do not provide information regarding the quality of the print structure [18,19].

A potential technology that can be used to provide structural information is machine vision (MV), which is a subset of artificial intelligence (AI). AI is an emerging technology in pharmaceutics in which machines are trained to perform tasks like humans, with the end goal of replacing human activity. In pharmaceutics, AI has the potential to accelerate developments through automating tasks of varying complexity, and has recently been applied to predicting 3D printing outcomes, food–drug interaction, crystallization, and the generation of nanoparticles [20,21,22,23,24]. Similarly, MV has been researched in pharmaceutics. MV, also referred to as computer vision, is a rapidly growing subset of AI that involves the use of digital images and pattern recognition algorithms to interpret and understand visual information. Compared to manual inspection, MV offers digital precision, speed, and is not subject to human error or bias [25]. In manufacturing, MV systems can be employed for real-time QC monitoring, allowing them to perform tasks such as inspection and identification [26]. By automating visual tasks and providing fast and accurate results, MV is helping to improve efficiency, reduce errors, and enhance safety in various applications [27,28,29,30]. In pharmaceutics, MV was found to achieve high accuracy in the real-time detection of measuring coating thickness and detecting defects in film-coated tablets [28]. It has also been used for inspecting liquid droplets in pharmaceutical injections [27], data augmentation [30], and in situ monitoring of the selective laser sintering process [29].

Collectively, these studies demonstrate that MV can perform automated QC tasks with high accuracy, precision, and speed, which could be applied to pharmaceutical 3D printing. However, a large dataset is required for model development, which for nascent technology like 3D printing, can be a time-consuming and costly endeavor [31]. One approach is to generate synthetic data that are used to train the AI platform. Synthetic data refers to data that are artificially generated to resemble real-world data. They are created using algorithms and models that consider patterns observed in existing data. Synthetic data are simpler and more cost effective to obtain than real data [32]. Additionally, synthetic data are more sustainable and can be generated in potentially unlimited numbers with pre-annotation, which would otherwise be required to be added manually to the real-world data [33]. Synthetic data have been successfully demonstrated to enhance model performance [31,32,33,34,35]. Additionally, it is important to highlight that the Medicines and Healthcare products Agency (MHRA) has introduced two synthetic datasets that have played a significant role in advancing medical technologies in the fight against COVID-19 and cardiovascular disease. According to Janet Valentine, the Director of the Clinical Practice Research Datalink, these synthetic datasets will assist in accelerating the market entry of safe products, thereby enabling patients to enjoy the advantages of cutting-edge technological progress [36]. Therefore, with its growing adoption, synthetic data could address the lack of data available in 3D printing.

In this study, synthetic data were used to train an AI pipeline that could be applied as a QC method of 3D-printed pharmaceuticals. The pipeline consisted of MV techniques to process the images, followed by machine learning (ML) for image classification. Synthetic data in the form of photorealistic rendered images were used to train ML models to distinguish between ‘Good’ and ‘Bad’ printing outcomes. The study focused on rendering images of three commonly printed dosage forms: capsules, tablets, and films. Once trained, the ML models were tested on real images of 3D-printed dosage forms, which were fabricated by stereolithography (SLA). The performance of the ML models was scrutinized, and the results are discussed herein.

## 2. Experimental Procedure

### 2.1. Virtual Image Generation

Virtual images (i.e., synthetic data) were generated by combining Python and Blender software, creating a streamlined pipeline to ensure consistency, replicability, and speed. The Blender software version used was v3.4 (Blender Foundation, Amsterdam, Netherlands), while the Python version used was Python v3.11 (Python Software Foundation). Three dosage forms were manually designed: films, tablets, and capsules. The design process aimed to create photorealistic and accurate representations of 3D-printed dosage forms. Detailed geometrical features were considered for each type of dosage form. The film design was 30 mm by 10 mm with a thickness of 1 mm. The tablet design was cylindrical with a diameter of 10 mm and height of 3 mm, and the capsule design was two-part cylindrical with smooth surfaces, with a length of 18 mm and a diameter of 8 mm.

**Figure 1:**
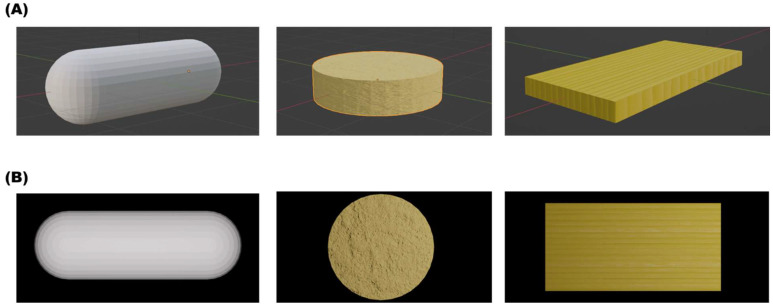
Representative images of (**A**) the models generated using Blender and (**B**) the top-view rendered image used for training the ML models. Attempts were made to capture the realism of 3D-printed dosage forms.

A Python script was developed to automate the process of generating different images from the original versions. This script incorporated a loop function to produce 100 ‘Good’ images for each dosage form. ‘Good’ images were defined as those containing a defect-free print. The ‘Bad’ images comprised three different subcategories, which were ‘over-cured’, ‘under-cured’, or ‘cracked’. A Python script was developed to loop through each subcategory to produce 33 images from each, and an additional image was manually added. Thus, there were 100 ‘Good’ and 100 ‘Bad’ images used for model training. The Python script was integrated with the Blender software using Blender’s Python application programming interface (bpy v3.4.0). This integration allowed for direct control over the Blender environment and objects via Python, enabling the color variations of the dosage forms to be systematically and consistently altered [37]. The camera, lighting, and print positions were kept constant throughout the loop. After each iteration of color alterations, the image was rendered and saved. Representative models generated by Blender are presented in forms (Figure 1).

### 2.2. SLA Image Generation

#### 2.2.1. Computer-Aided Design (CAD) of Formulations

Onshape (PTC, Boston, MA, USA) was employed to design the 3D model of all formulations in this study. The diameter of the tablets was 10 mm, and their thickness was 3 mm. The capsules measured 18 mm in length and had a diameter of 6 mm. The films had a length of 20 mm, a width of 10 mm, and a thickness of 0.5 mm. Meanwhile, three different types of ‘Bad’ prints, or printing deformities, were intentionally created for each formulation, including ‘cracked’, ‘under-cured’, and ‘over-cured’.

#### 2.2.2. SLA Printing Process

**Figure 2:**
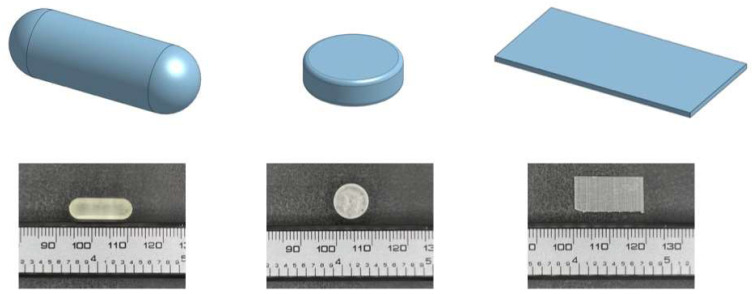
3D visualizations displaying CAD models of dosage designs (capsule, tablet, film) alongside their corresponding SLA prints, captured by a digital camera. These visualizations provide insights into the design-to-reality translation and highlight key features of each design.

Dosage forms were printed using a commercial Form 2 SLA 3D printer (Formlabs Inc., Milbury, OH, USA) that was equipped with a 405 nm laser. A similar SLA pipeline to one that was previously published was adopted herein [38]. The CAD model was exported as a stereolithography (.stl) file and then uploaded to the Preform Software v.3.28.0 (Formlabs Inc., Milbury, OH, USA), where necessary adjustments and support structures were added before printing. The formulations were printed on the building platform through the polymerization of the photocrosslinkable resin (Clear V4, Formlabs Inc., Milbury, OH, USA) with a layer thickness of 0.1 mm. The prints were then washed in isopropanol for 2 min to remove any remaining resin on the surface. Afterwards, Form Cure (Formlabs Inc., Milbury, OH, USA) was employed for the post curing process at 60 °C for 45 min under a light source (λ = 405 nm). The dosage forms were successfully prepared after the removal of support structures with a diagonal cutter. Examples of the CAD model and 3D-printed dosage forms can be viewed in Figure 2.

#### 2.2.3. Photogrammetry Setup

**Figure 3:**
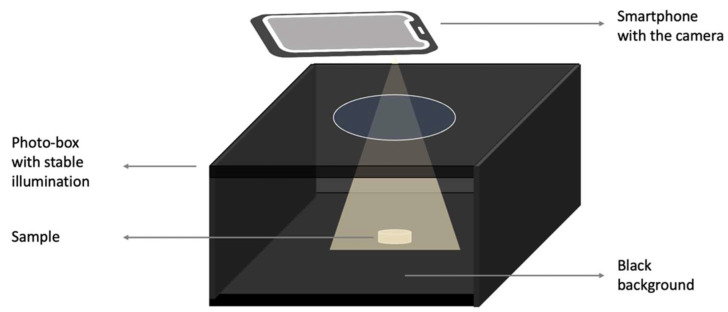
Schematic diagram depicting the photogrammetry setup.

A photogrammetry setup was used to help improve the quality of the image acquisition, which provides a control source of light, as well as ensuring reproducibility [39]. Image acquisition was completed in a commercial photo-box purchased from Amazon. It was equipped with an LED panel, which provided a stable and evenly distributed illumination from top to bottom with a light intensity of 2912 Lumen (10 W). All images were captured from a top-down perspective using the ultrawide camera of an iPhone 13 Pro (Apple Inc., Cupertino, CA, USA) with a resolution of 12 million pixels. The distances between the camera and background are 4.5, 5.5, 6.5 cm for tablets, films, and capsules, respectively. A schematic of the setup can be seen in Figure 3.

### 2.3. AI Pipeline Development

The software and libraries used for the AI pipeline were as follows: Python v3.11 (Python Software Foundation), scikit-learn (sklearn) v1.2.1, and OpenCV v4.7.0. Python served as the primary programming language for scripting the pipeline due to its wide array of support for ML libraries and its extensive, easy-to-use syntax. The scikit-learn library was chosen for its comprehensive selection of both supervised and unsupervised learning algorithms and model evaluation tools [40]. OpenCV was used for its MV capabilities in image processing, which were crucial for pre-processing steps in the pipeline [41]. Prior to model training, the images underwent pre-processing using OpenCV. This pre-processing step was important for ensuring consistency across all images and improving the model’s performance. The pre-processing included image resizing to create uniformity in dimensions across all images and converting the images to grayscale to eliminate potential color-related discrepancies and reduce computational demand [42]. Once pre-processing was complete, the processed images were used as inputs for the ML model. The models used and the parameters tuned via a gridsearch are detailed in Table S1 [43]. Model performance was evaluated using the accuracy, specificity, sensitivity, and area under the receiver operating curve (AUROC) metrics. Furthermore, the Brier score and probability distributions were calculated and plotted to assess confidence in the model’s predictions. The formulae for the metrics are presented below [44]:

(1) $$Accuracy=\frac{\left(True\;Positives+True\;Negatives\right)}{\left(True\;Positives+False\;Positives+True\;Negatives+False\;Negatives\right)}$$

(2) $$Sensitivity=\frac{True\;Positives}{\left(True\;Positives+False\;Negatives\right)}$$

(3) $$Specificity=\frac{True\;Negatives}{\left(True\;Negatives+False\;Positives\right)}$$

Brier Score = (1/N) × Σ (predicted probability − actual)^2^ (4)

where, N is the total number of predictions; predicted probability is the probability that was predicted; and ‘actual’ is the actual outcome (‘Good’ or ‘Bad’). The lower the Brier score, the better the performance of the prediction model. AUROC is a plot of true positive rate (sensitivity) against false positive rate (1-specificity) at various threshold settings. The area under this curve quantifies model performance; an area of 1 represents a perfect test, while an area of 0.5 represents a worthless test [45].

## 3. Results

Virtual images of capsules, tablets, and films were generated on a computer (Figure 1). A total of 100 images were generated for each dosage form, with varying color and textures to add variety. A further 100 images representing prints with defects were also virtually generated to teach the model the difference between a successful 3D-printed product and a product with a defect common to SLA printing [46,47,48,49,50].

### 3.1. Exploratory Data Analysis (EDA)

EDA serves as a crucial preliminary step in ML, providing the opportunity to examine data prior to feeding them into a model. It can identify potential anomalies that can then be addressed before the model development process and directs subsequent data handling. Given that the input data for this study were images, various pre-processing measures could be implemented, making a preliminary data inspection beneficial in deciding which methods to adopt [51,52].

**Figure 4:**
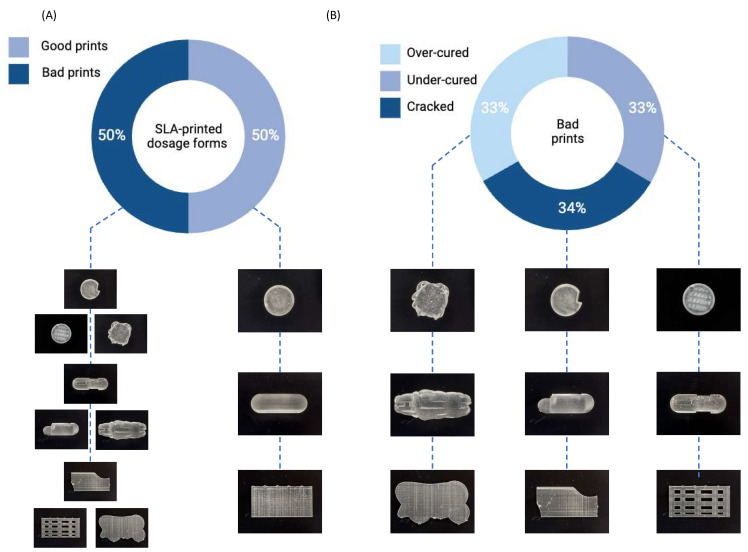
Donut charts depict (**A**) the ratio of good and bad SLA-printed dosage forms, and (**B**) the ratio of defects within the bad prints.

For this preliminary study, the ratio between ‘Good’ and ‘Bad’ prints was kept at 50:50 for each dosage form to prevent model bias (Figure 4A). Similarly, a total of 200 images for each SLA-printed dosage form were captured using a smartphone, also with a ratio of 50:50 between ‘Good’ and ‘Bad’ quality, to form the testing dataset. For the ‘Bad’ SLA prints, defects were purposefully introduced in the CAD model. Among the bad quality forms, approximately one-third were produced to replicate over-curing, one-third to replicate under-curing, and the remaining one-third were produced to replicate cracked prints (Figure 4B).

**Figure 5:**
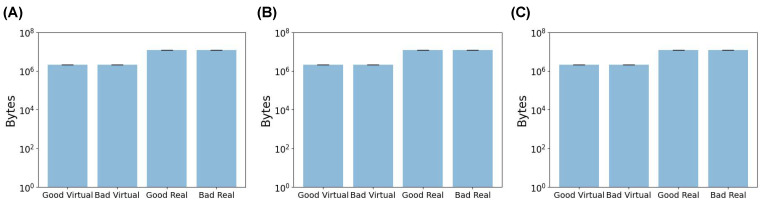
The mean file size for (**A**) capsules, (**B**) tablets, and (**C**) films in bytes.

The image size for both the virtual images and real images was also inspected. In the case of the virtual images, all three dosage forms had image sizes of about 10^6^ bytes, with a narrow distribution demonstrated by the small error bars (Figure 5). Conversely, the smartphone-captured SLA images were approximately 10^7^ bytes in size and exhibited a compact distribution (Figure 5). Therefore, there was a ten-fold difference between the sizes of the virtual and real images. ML models require input data with consistent dimensions, implying that both the training and testing datasets must possess the same feature vector length. Therefore, pre-processing was necessary to ensure both sets of images aligned to the same dimensional scale.

The color distribution of both image sets was also investigated. Several color spaces can be explored, with the RGB color space, representing red, green, and blue, being most common. These three colors together represent a wide spectrum of hues. Within the RGB space, the intensity varies from 0 to 255, with higher values indicating greater color intensity.

**Figure 6:**
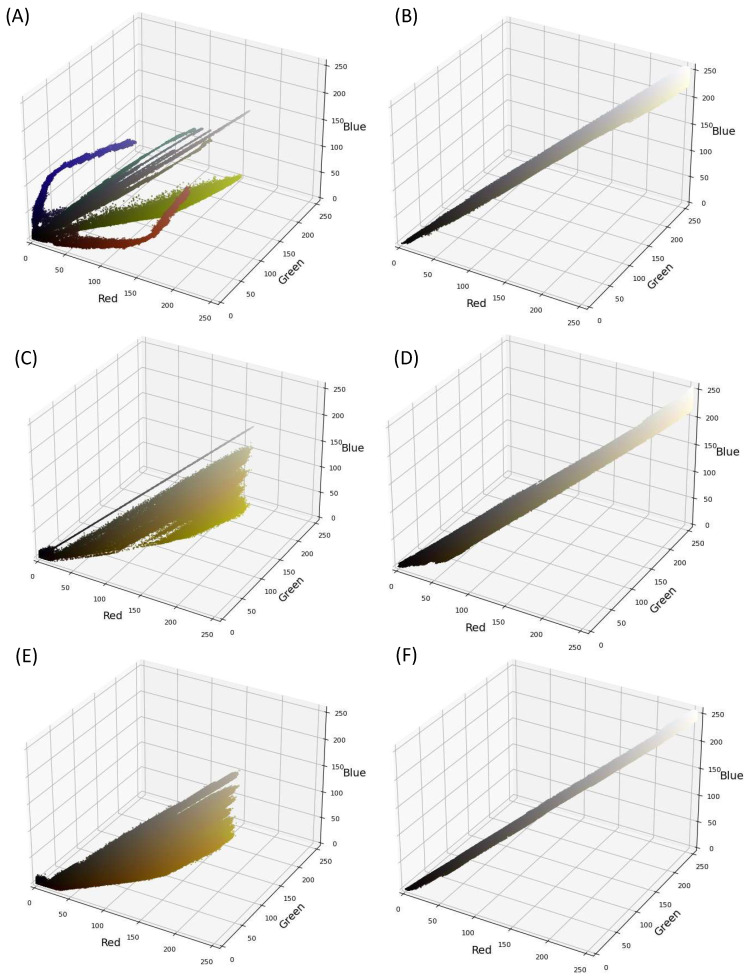
Color distribution in real images (**A**,**C**,**E**) and in virtual images (**B**,**D**,**F**) of capsules (**A**,**B**), tablets (**C**,**D**), and films (**E**,**F**), respectively.

Upon examining the color distribution, the virtual images were found to exhibit a wider color distribution with shades of black, yellow, orange, and white, as evidenced in Figure 6A,C,E. The virtual capsule images possessed additional colors, as depicted in Figure 6A. In contrast, the real images exhibited a limited color gradient, spanning between black and white (Figure 6B,D,F). This signifies a potential color disparity between the two datasets. Color discrepancies are frequent in MV applications, and one common MV pre-processing strategy to rectify this involves converting the images to grayscale [42]. The grayscale color space is more constrained, with each pixel appearing in a single shade (i.e., grey) and an intensity ranging from 0 to 255.

### 3.2. ML Results

Seven ML techniques (MLTs) were investigated for this task. Each one was trained on the 200 virtual images and subsequently asked to predict the quality of the actual SLA-printed dosage form. Untuned models were initially investigated, however, they were unable to achieve accuracies above 50%. This indicated that the models were at best randomly classifying the images, and hence not actually learning from the virtual images. Subsequently, the models were then tuned with their respective hyperparameters (Table S1), and the results are presented below.

#### 3.2.1. The Effect of Image Size

**Figure 7:**
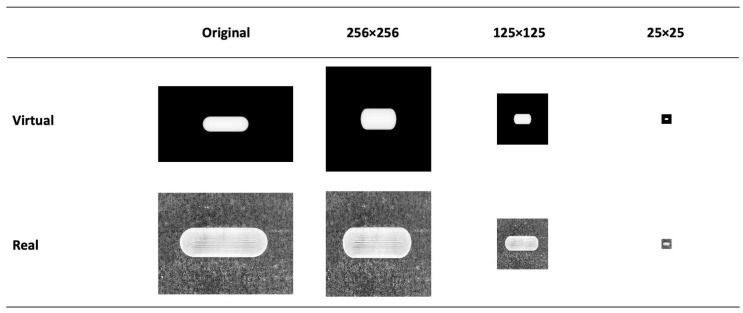
Example of the effect of image re-sizing on the dosage form structure.

The EDA revealed that the two image datasets had different image sizes and were substantially large. For this reason, their image sizes were reduced to a smaller scale, ranging from 25 × 25 to 512 × 512 pixels. It is essential to note that after resizing, both datasets had the same scale, making them compatible for ML processing. We reiterate that it is critical for these ML models to have consistent feature sizes between training and testing datasets. For instance, if the training data have five variables and the testing data have six, the model will not function correctly. While resizing images does remove pixels, our study found that the overall image structural integrity was largely maintained, as illustrated in Figure 7 and Figures S1 and S2.

**Figure 8:**
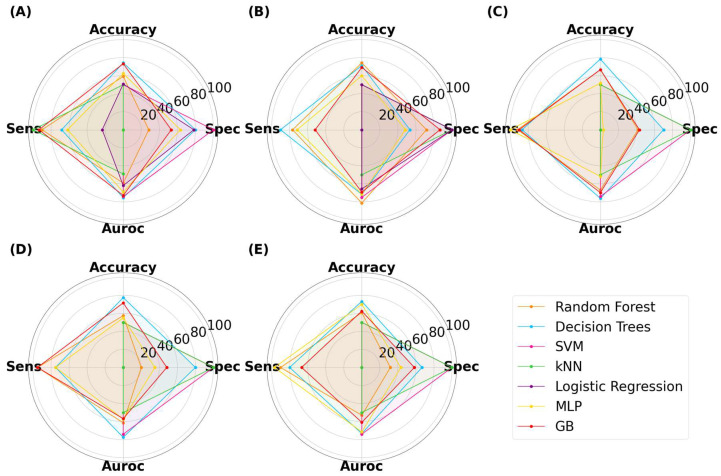
MLTs’ performance for classifying SLA-printed capsules, with image sizes of (**A**) 25 pixels, (**B**) 50 pixels, (**C**) 125 pixels, (**D**) 256 pixels, and (**E**) 512 pixels.

The ML results for capsules are presented in Figure 8. The classification accuracy reached as high as 78.3%, indicating that ML models trained on virtual images can predict 3D-printed capsules. The highest accuracy was attained by decision trees (DT), with an image size of 125 × 125 pixels. The same model had a specificity, sensitivity, and AUROC value of 70.0%, 87.0%, and 76.2%, respectively. Thus, DT presented with high performance across all metrics. Despite the reduction in image size, ML models were able to achieve high performance. There was no correlation between the image size and model classification performance between 25 × 25 to 512 × 512 pixels. The second-best performing combination was a DT with a size of 256 × 256 pixels, where the accuracy was 77.5%. Random forest (RF) is an ensemble of decision trees and has been found to outperform a single decision tree in complex tasks [53,54]. The highest accuracy attained by RF was 74.2% with an image size of 50 × 50 pixels. Interestingly, RF performed better when it used a low number of decision trees (data not provided). Gradient-boosted decision trees (GB) is another tree-based algorithm that has been found to perform well in classification tasks [55]. Its maximum accuracy was 73.3% with an image size of 25 × 25 pixels. Multi-layer perceptron (MLP) is distinctively different to tree-based models and learns by a series of neuron layers. Its highest accuracy was 70.0% with an image size of 512 × 512 pixels. Support vector machines (SVM) and k-nearest neighbor (kNN) have distinct learning characteristics compared with the aforementioned MLTs, but these models were consistently found to exhibit low classification accuracy. Logistic regression (LR), being the only linear model used herein, exhibited accuracies of 50%, despite being tuned. Moreover, the computational demand for LR exceeded the limits available when training was performed on image sizes of 125 pixels and above. Nonetheless, the results indicated that linear models were performing equivalent to random chance.

**Figure 9:**
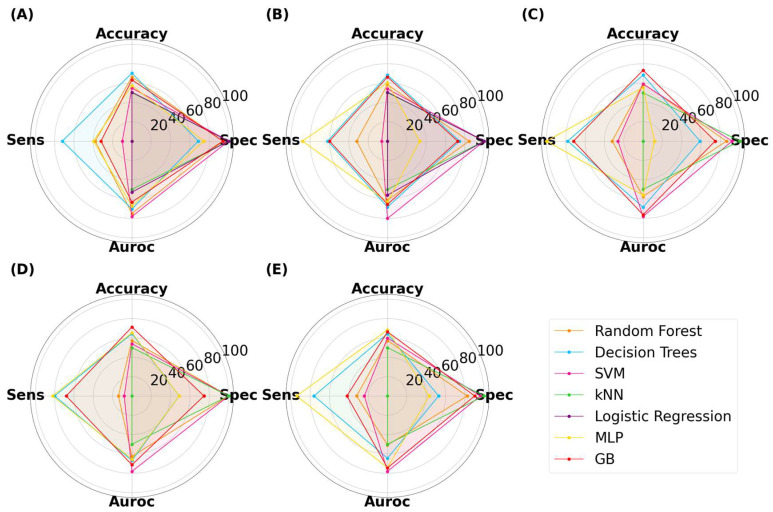
MLTs’ performance for classifying SLA-printed tablets, with image sizes of (**A**) 25 pixels, (**B**), 50 pixels, (**C**) 125 pixels, (**D**) 256 pixels, and (**E**) 512 pixels.

Similar relationships were observed with ML models classifying the SLA-printed tablets (Figure 9). The highest performing model was GB, which achieved an accuracy, specificity, sensitivity, and AUROC of 73.3%, 75.0%, 72.0%, and 76.2%, respectively, with an image size of 125 × 125 pixels. It was noticed that the computational demands increased with image size, with LR exceeding the limits at image sizes of 125 pixels and above, and it was thus unable to complete the task. At lower image sizes, LR again achieved a maximum accuracy of 50%, once again indicating that it was randomly guessing. DT also performed well, with the highest accuracy being 70.3% at 25 × 25 pixels. Interestingly, neither RF nor MLP achieved an accuracy of 70% or above, indicating that both models performed less well for tablets than with capsules.

**Figure 10:**
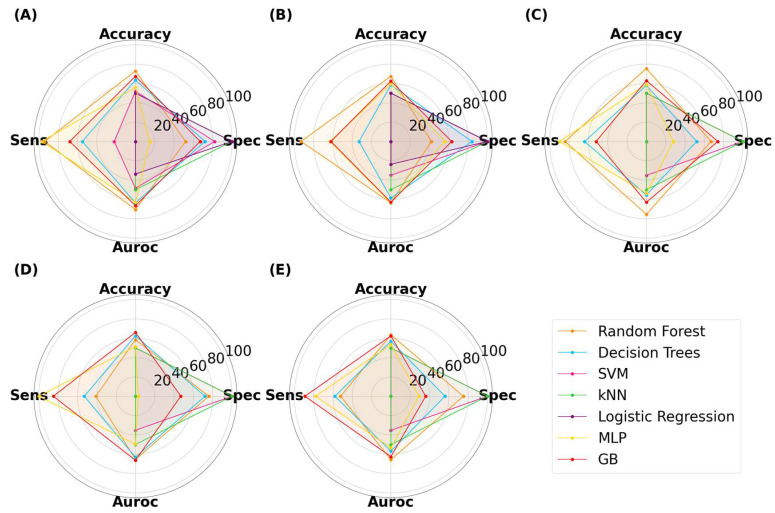
MLTs’ performance for classifying SLA-printed films, with image sizes of (**A**) 25 pixels, (**B**) 50 pixels, (**C**) 125 pixels, (**D**) 256 pixels, and (**E**) 512 pixels.

For SLA-printed films, the highest performance was achieved by RF, with an accuracy, specificity, sensitivity, and AUROC of 75.5%, 67.0%, 84.0%, and 75.5%, respectively (Figure 10). The results show that RF was able to achieve a balance between all metrics. LR was again limited to 50% accuracy, demonstrating that non-linear models perform better than linear models for this task [56].

#### 3.2.2. The Effect of Grayscale Transformation

The EDA revealed a disparity in color gradient between the virtual and real images (Figure 6). Thus, grayscale transformation was implemented to determine if this could improve model accuracy. The analysis with varying image sizes was repeated and the results are displayed in Figures S3–S5. For capsules, the highest performing model was GB with an accuracy of 80.8% at 512 × 512 pixels. The specificity and sensitivity were relatively balanced at 77.0% and 85.0%, respectively. The AUROC was also high at 83.3%, which indicated that the model was able to accurately differentiate between the two classes. Both DT and RF were able to achieve accuracies above 75% and with a relatively acceptable balance between specificity and sensitivity. As grayscale has fewer dimensions than the RGB space, model development was noticeably faster. Moreover, LR was able to make predictions at image sizes up to 125 × 125 pixels, having previously exceeded computational limits at this pixel size. The highest accuracy by LR was 62.5%, which was at 125 × 125 pixels. For the grayscale tablets, the highest accuracy of 74.3% was achieved by DT at 50 × 50 pixels, with the specificity and sensitivity being 84% and 64%, respectively. For the grayscale films, the best performing model was also DT at 512 × 512 pixels, with an accuracy, specificity, sensitivity, and AUROC of 71%, 56%, 86%, and 71%, respectively. For both grayscale tablets and films, the accuracy for LR was 50%. Overall, it was evident that grayscale transformation was not detrimental to model development, regarding both accuracy and computational demands. Moreover, it was evident that tree-based models performed better than the other learners for classifying the quality of dosage forms.

### 3.3. Model Robustness: Stress Testing to Ensure High Performance

#### 3.3.1. Scalability

The results thus far were encouraging and warranted further stress testing to ensure the ML models’ robustness. The first stress test was to determine model scalability. The test image dataset (i.e., real SLA images) was augmented from 200 to 3200 images. Augmentation, which is a common MV method, introduces varied transformations to images, such as rotation or brightness adjustments, to emulate real-world data variations and increase dataset breadth [57]. This process ensures the model’s genuine adaptability and not just its familiarity with the initial dataset’s specific patterns. By evaluating performance on augmented data, potential overfitting can be detected, where the model might excel with original images but falter with transformed images. Furthermore, testing with augmented images mimics unpredictable real-world scenarios, thereby providing a more robust assessment of the model’s practical utility. This comprehensive approach boosts confidence in the model’s consistent performance, highlighting its adaptability and readiness for deployment across varied scenarios.

**Figure 11:**
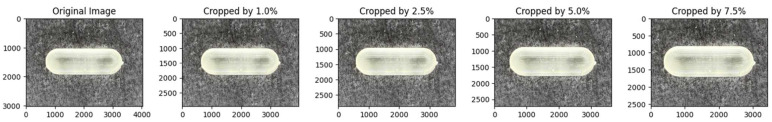
Representative images demonstrating the effect of cropping. Varying the cropping size can mimic the imaging distance. Increasing the cropping amount made the dosage form appear larger.

**Figure 12:**
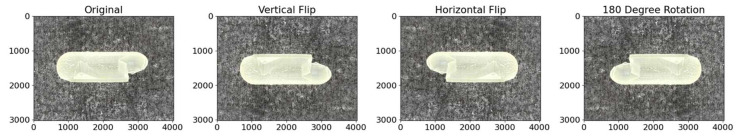
Representative images demonstrating the effect of a vertical and horizontal flip, and a 180-degree rotation. From these transformations, new images can be generated to mimic, for example, different cracked locations on the capsule.

In this study, the testing dataset was augmented by first transforming the images to grayscale and then cropping the images by four different values (Figure 11). The training images were inspected following cropping to ensure that it did not affect the dosage form. Cropping simulates the potential imaging distance between the object and the camera. Following cropping, the training images were then flipped both horizontally and vertically, and rotated at 180°, which simulated different imaging orientation of the dosage forms (Figure 12). With these transformations, the testing dataset grew from 200 to 3200 images. In the interest of time, only DT and RF models were explored, as they were found to consistently produce high accuracies and exhibited low computational demands. The training dataset was only transformed to grayscale to ensure that it had the same vector space as the training set, and as such, remained at 200 images. As before, the effect of image size was also explored.

**Figure 13:**
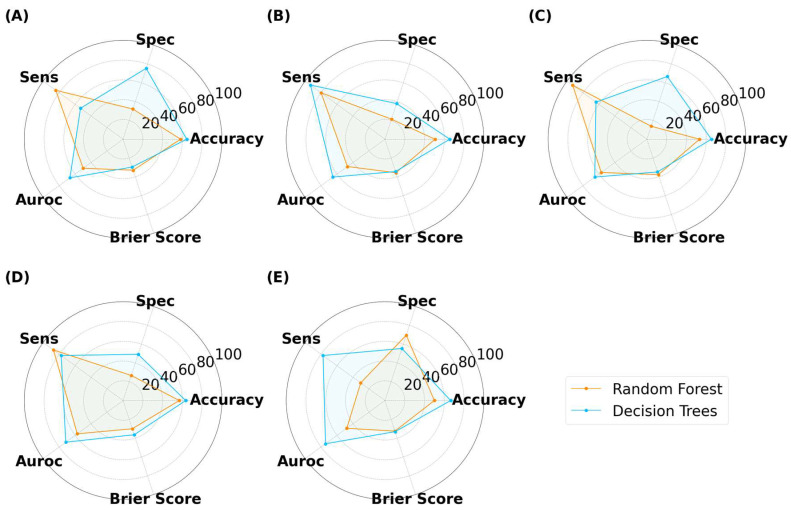
MLTs’ performance for classifying SLA-printed capsules after data augmentation, with image sizes of (**A**) 25 pixels, (**B**) 50 pixels, (**C**) 125 pixels, (**D**) 256 pixels, and (**E**) 512 pixels. (Note, the Brier score is on a scale from 0 to 1).

**Figure 14:**
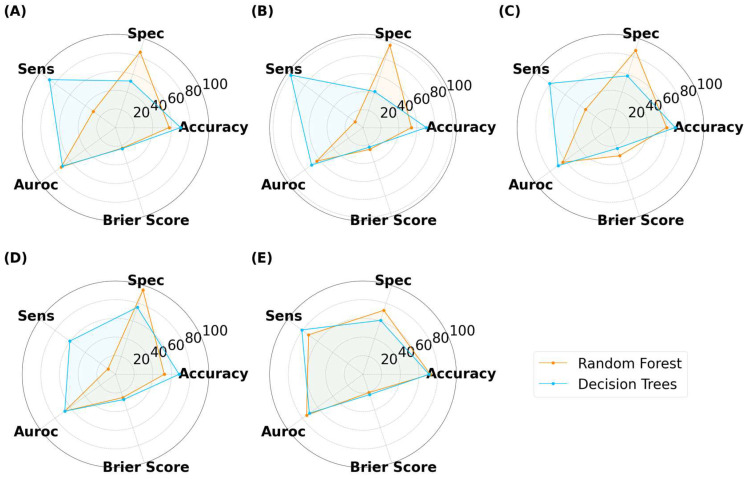
MLTs’ performance for classifying SLA-printed tablets after data augmentation, with image sizes of (**A**) 25 pixels, (**B**) 50 pixels, (**C**) 125 pixels, (**D**) 256 pixels, and (**E**) 512 pixels. (Note, the Brier score is on a scale from 0 to 1).

**Figure 15:**
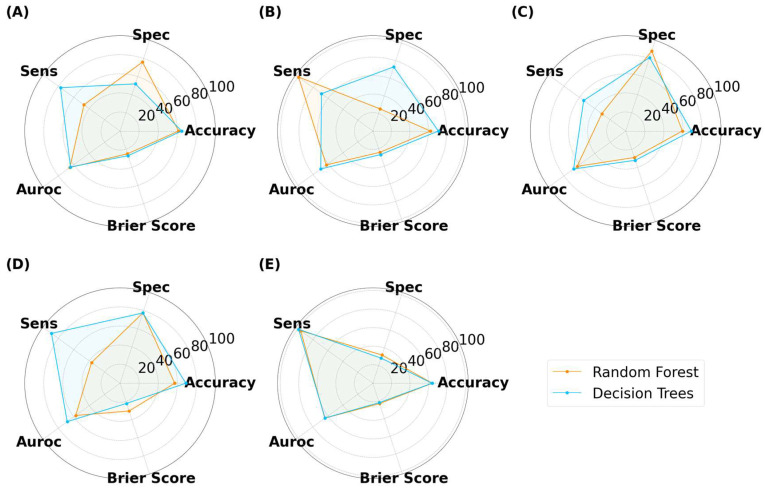
MLTs’ performance for classifying SLA-printed films after data augmentation, with image sizes of (**A**) 25 pixels, (**B**) 50 pixels, (**C**) 125 pixels, (**D**) 256 pixels, and (**E**) 512 pixels. (Note, the Brier score is on a scale from 0 to 1).

As illustrated in Figure 13, Figure 14 and Figure 15, the models were able to maintain their high performance, with the highest accuracies for capsules, tablets, and films being 66.5%, 72.0%, and 70.9%, respectively, that were achieved by DT, RF, and DT, respectively. Therefore, the results show that model performance can be scaled to 3200 new imaging instances. For the augmented capsule test dataset, DT outperformed RF at all image sizes, with accuracies ranging from 63.3% to 66.5%. The image sizes that gave the highest accuracy of 66.5% for DT were the 512 × 512 pixel images, which also had the highest AUROC value of 74.4%. A higher AUROC value infers that DT could achieve a higher accuracy by adjusting the classification threshold. It also reassures that DT performs better than randomly guessing [58]. The accuracies for RF ranged from 50% to 58.2%. RF produced high sensitivity values, indicating that it was good at recognizing the true images (i.e., ‘Good’ images). However, its specificity for 4/5 image sizes was less than 50%, indicating that RF struggled to classify negative images (i.e., to recognize bad images as only ‘Bad’).

For tablets, DT achieved higher accuracies than RF in all but the 512 × 512 pixels (Figure 14). The DT models tended to have a greater sensitivity than specificity for tablets, indicating that the models performed well at distinguishing true ‘Good’ tablets. In contrast, RF exhibited a balance between specificity (72.1%) and sensitivity (72.0%) at images of 512 × 512 pixels. However, this balance was lost at lower image sizes, with RF exhibiting notably higher specificity than sensitivity. This infers that image sizes of 256 pixels or lower were unable to recognize true ‘Bad’ tablets. RF with image sizes of 512 × 512 pixels were the best performer for the scaled tablet datasets, with an accuracy and AUROC of 72.0% and 74.6%, respectively. The higher AUROC inferred that the model was good at distinguishing between ‘Good’ and ‘Bad’ tablets.

For films, DT presented with marginally higher accuracies than RF (Figure 15). In contrast to classifying capsules and tablets, preference for specificity and sensitivity varied with image size for both models. For example, RF presented higher specificity for image sizes of 25 × 25 and 125 × 125 pixels, but higher sensitivity for 50 × 50 and 512 × 512 pixel images. The best performer was DT with an image size of 50 × 50 pixels, where the accuracy and AUROC were 70.9% and 70.0%, respectively. Hence, maintaining the classification threshold at 0.5 was sufficient in achieving good accuracy. The collective results for capsules, tablets, and films demonstrated that training ML models on virtual images was scalable to a larger number of images, however, the MV pipeline for each solid dosage form will need to be separately optimized.

#### 3.3.2. Model Confidence

To ensure the rigorousness of the methodology in developing the ML model, the study went beyond conventional accuracy metrics and examined the confidence of the models’ predictions. The Brier score was leveraged for this purpose, serving as a critical measure of the models’ confidence in its predictions. The Brier score is a unitless metric, ranging from 0 to 1, where a lower value indicates greater confidence and calibration of the models’ probabilistic classification [59]. The emphasis on confidence is pivotal because it offers a deeper understanding of the model robustness. A model that exhibits high variance in confidence levels might produce inconsistent predictions when re-trained, posing challenges for reproducibility and reliability. The Brier score, with its ability to quantify the difference between predicted probabilities and actual outcomes, becomes a necessary tool to assess such inconsistencies. While the benchmark for an ‘ideal’ Brier score can vary based on the specific application, a general guideline is that scores below 0.25 tend to indicate acceptable confidence levels, strengthening assurance in the model’s robustness and reliability [59].

**Figure 16:**
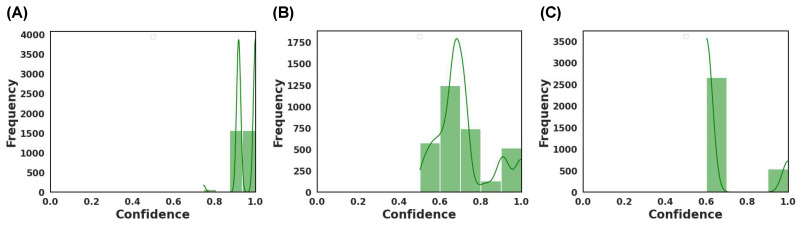
Model confidence for the best performing MLT for (**A**) capsule achieved by DT, (**B**) tablets achieved by RF, and (**C**) films achieved by DT.

For all three dosage forms, the Brier score ranged between 0.20 to 0.40 (Figure 13, Figure 14 and Figure 15). For the aforementioned models with the highest accuracy, their respective Brier scores were 0.24 (capsule), 0.21 (tablet), and 0.27 (film). Hence, while the DT obtained the highest accuracy for classifying film quality, its Brier score indicates that it was marginally worse than a model that predicts with complete uncertainty. To obtain further insight, the respective probability distributions are presented in Figure 16, in the form of most confident by applying Equation (5). Here, the values are scaled from 0.5 (low confidence) to 1.0 (high confidence):

(5) $$most\;confident={{max}_{\hat{y}}\;P}_{\theta }\;\left(\hat{y}|x\right)$$

where ${{max}_{\hat{y}}\;P}_{\theta }\;\left(\hat{y}|x\right)$ is the maximum conditional class probability given the input features. In other words, the formula returns the probability for the predicted class, which makes it easier to visualize the probability distribution.

As depicted, the models varied in their confidence (Figure 16). DT was found to be the best performing model for classifying capsules and films, where the most confident values ranged from 0.75 to 1.0 and 0.6 to 1.0, respectively. Hence, DT was generally more confident in predicting capsules than films. RF, which performed best for classifying tablets, had a wide confident distribution between 0.5 and 1.0, with the mode around 0.7. The wide distribution infers that the model was confident in predicting some images and less confident with others. Thus, when combined, the results of the Brier score and the most confident ranges indicated that the models exhibited a range of confidence in their predictions.

#### 3.3.3. Out-of-Data (OOD) Distribution

**Figure 17:**
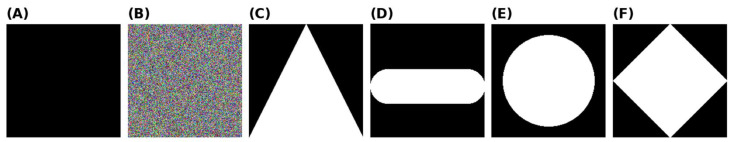
Samples of the out-of-data distribution images are used to stress test the models: (**A**) black background, (**B**) random noise, (**C**) triangle, (**D**) oblong, (**E**) circle, and (**F**) diamond shape.

The final stress test was to observe the models’ performance for detecting random or irrelevant objects. The primary objective was to ensure that the model would not misclassify random objects or images as ‘Good’. Both a hyperparameter-tuned and untuned DT and RF were evaluated for their ability to classify four different types of images, of which examples are presented in Figure 17. These samples were purposely selected to highlight specific features that the model might be recognizing during training. Each model was fed 200 examples of each of the four different models, and their accuracy was evaluated.

**Figure 18:**
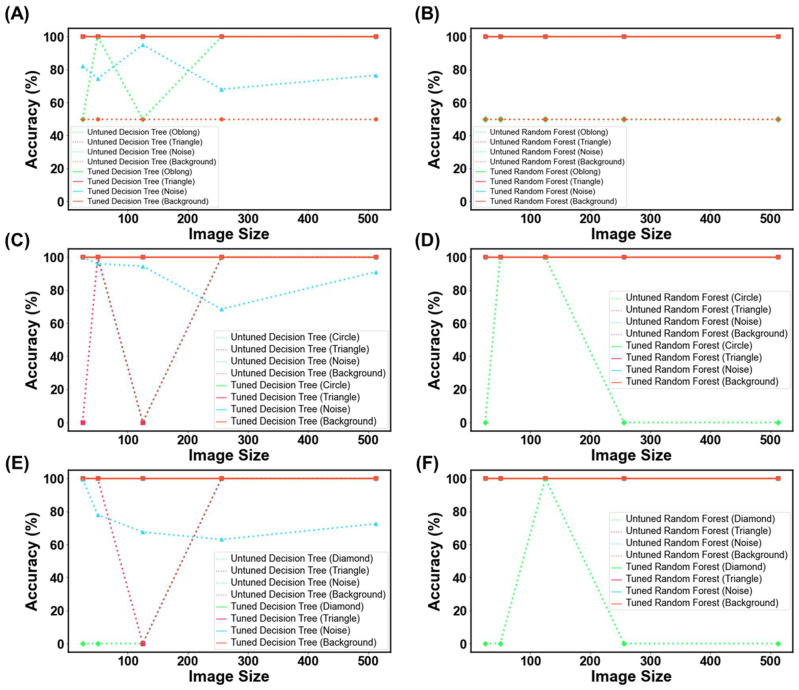
MLT metrics when tested on the out-of-data distribution for (**A**,**B**) capsule, (**C**,**D**) tablet, and (**E**,**F**) film. The purpose of this test was to determine how DT and RF would perform when presented with irrelevant images.

For untuned DT and RF trained with virtual capsule images, both models struggled with recognizing the plain background as not a capsule, with both models presenting with a 50% classification accuracy (Figure 18A,B). When tested on randomly generated pixels (referred to herein as noise image), the untuned RF was found to be robust against the noise, whereas DT was affected by the noise, where it was evident that DT was classifying the noise images as ‘Good’ capsules. When tested on the triangle images, both models were robust to this geometry, and thus did not classify any of these images as ‘Good’. However, when tested with oblong geometry, RF was classifying 50% of oblong images as ‘Good’ capsules across all image sizes, whereas DT had 100% accuracy for all image sizes but 25 and 125 pixels. Thus, the results infer that the untrained model could potentially recognize oblong objects in an image as a capsule. This could explain why previously untuned models struggled to achieve accuracies above 50% in the previous task (3.2. ML Results). Fortunately, when the models were hyper-parameter tuned, they performed perfectly and were able to recognize that none of these OOD images were a real capsule.

Similar trends were also seen for the other two dosage forms, whereby untuned models struggled with classifying these OOD images, and similarly, their tuned counterparts were robust against them (Figure 18C–F). The ultimate test was to discern if the model might mistakenly categorize images of similar geometric shapes, like oblong for capsules, circles for tablets, and diamonds for films (both being quadrilateral shapes). Both the tuned DT and RF were able to accurately classify these images. This indicated that the models were not overfitting for the ‘Bad’ class and were able to recognize other types of objects as bad. In other words, the models were able to recognize that ‘Bad’ prints could exist beyond just cracked, over-cured, and under-cured films. Equally impressive, the results suggest that the models are tuned to expect features beyond geometric patterns with edges, inferring that they are looking for other features that make a good SLA-printed dosage form.

## 4. Discussion

The study demonstrates the utility of using virtual images for training ML models, and by extension, addresses the issue of data shortage in pharmaceutical 3D printing. As mentioned, virtual images have been used outside of pharmaceutics for AI modelling [60,61,62,63,64,65]. Moreover, virtual reality has been proposed to train researchers, offering a number of benefits centered around economic, environmental, and human safety sustainability [66]. Collectively, these studies allude to the virtual domain playing a key part in revolutionizing the future of pharmaceutical research.

The unexpectedly high model accuracy attained herein can be explained by the use of photo-realistic images, capturing textures such as layers that are commonly seen in 3D-printed dosage forms (Figure 1). The results presented in Figure 18 verify this by illustrating that the models are learning features from the images beyond simple geometric outlines. The utility of virtual images was also demonstrated by purposefully selecting a balanced dataset, where an imbalanced dataset has been known to hinder model performance [67]. Credit should also be given to MLTs for the high accuracies attained and their ability to establish non-linear, complex relationships in high-dimensional data. In a prior study, a deep learning model was able to achieve an accuracy of 98.24% in identifying coating defects from captured tablet images. This remarkable performance can be attributed to their utilization of a publicly available pre-trained deep learning model, as well as their adoption of a methodology that allowed for a greater tolerance of errors in defining correct predictions. Furthermore, the study involved the production of approximately 640 tablets for the purpose of training the model, with defects manually introduced to these tablets [28]. Incorporating virtual images into the training process could have rendered it more cost effective, less labor intensive, and environmentally sustainable.

The models were stress tested due to the large complexity of the task. For example, an image size of 512 × 512 pixels equates to 262,144 features per image, which is considerably larger than any feature size previously used [21]. Considering that every MLT will inspect every pixel in an effort to learn the difference between a ‘Good’ and ‘Bad’ print, there was potential for the MLTs to focus on the wrong pixels, and hence why the models were tested with the images from Figure 18 [68]. Moreover, feature sizes in the order of thousands and with only 200 training instances could have resulted in models producing high accuracies but lacking confidence in their predictions [69]. The difference between accuracy and confidence is that accuracy refers to how often a model’s predictions are correct compared with the actual outcomes, while confidence indicates the model’s own belief in the reliability of its predictions. Hence, the study went above and beyond to measure the Brier score and inspect the probability distribution. More work will indeed be needed to see if the current approach can reach the 98% accuracies attained in previous work [28].

Varying the image size between 25 × 25 to 512 × 512 pixels resulted in varying model performance, with no obvious trend. This could be due to the minor distortions during the re-sizing of pixels. For example, some resizing values for films were found to affect their aspect ratio, where the films changed from rectangle and square and tablets became elongated (Figures S1 and S2). Future work will seek to develop either a photogrammetry set that will account for these distortions or implement an MV pre-processing technique to avoid these distortions. On the other hand, grayscale transformation was found to have no significant impact, inferring that color was not a key feature for the ML in distinguishing between ‘Good’ and ‘Bad’ dosage forms.

As ML is still an emerging field, the theorem of no free lunch still applies here and thus several MLTs were explored [70]. Broadly speaking, the non-linear models behaved better than the linear models for this task. Further insight revealed that the DT was among the better performers where decision trees are considered to be simple algorithms. Upon further inspection of RF, the models that achieved the higher predictions used a fewer number of trees (<10 trees), which infers that using more trees results in overfitting [71].

Evidently, virtual images and other synthetic data can address the issue of lack of data available for ML model development that was previously mentioned [72]. Thus, virtual images are expected to accelerate developments yet maintain economic, environmental, and safe sustainable practices. This will indeed be helpful in helping 3D printing realize its industrial and clinical translational goals. Going forward, more work is needed to realize the extent of virtual images for developing sustainable ML models. It would be interesting to see how it performs on dosage forms produced by other 3D printing technologies. It would also be interesting to see how it performs on more complex dosage forms, like microneedles, and perhaps roll it out to the rest of the pharmaceutics field. In doing so, we can ensure that ML models are developed in a sustainable manner.

Although this study has demonstrated the utility of virtual images in training ML models manifesting remarkable performance in QC for 3D-printed dosage forms, it is imperative to discuss limitations that could impact the validity of the inspection. For instance, the ML models in this study were exclusively trained and tested on top-down view images, potentially overlooking structural defects that might only be observed from other perspectives. Therefore, ensuring the validity of predictive results may require the inclusion of images captured from different angles. Additionally, this research did not include the examination on the internal structure of dosage forms. Given the additive nature of 3D printing, it is plausible for defects to exist internally, even if the surfaces of the printed formulations appear intact. If our trained model were to examine images of dosage forms with internal defects, it would likely yield a relatively high false positive rate. Moreover, the pipeline looks at structural defects and not dimensional discrepancies. The pipeline’s utility could be expanded if it could detect dimensional discrepancies, as dimensional changes can affect content uniformity [73].

## 5. Conclusions

The study successfully demonstrated that virtual images of 3D-printed dosage forms can be used to train an AI model to recognize the quality of three different SLA-printed dosage forms. The highest recorded accuracies for classifying capsules, tablets, and films were 80.8%, 74.3%, and 75.5%, respectively. Tree-based models of DT and RF were found to consistently achieve high accuracies and were subsequently further stress tested against noisy and ‘distracting’ images. The stress tests revealed that the models were robust to these OOD images and were confident in their predictions of some tasks, obtaining Brier scores ranging from 0.20 to 0.40. Moreover, model deterioration was not observed when the models were tested against 3200 images augmented from the original SLA real images, demonstrating the pipeline’s potential for scaling to a larger image dataset. Therefore, it was concluded that virtual images hold great potential in training ML models for classifying 3D-printed dosage forms. Future work will seek to investigate the feasibility of the proposed pipeline to dosage forms generated by other 3D printing technologies.

## Data Availability

The data presented in this study are available on request from the corresponding authors.

## Supplementary Materials

The following supporting information can be downloaded at: https://www.mdpi.com/article/10.3390/pharmaceutics15112630/s1, Table S1: MLTs and their parameters for grid search, Figure S1: Representative images of tablets following re-sizing, Figure S2: Representative images of films following re-sizing, Figure S3: MLTs performance for classifying SLA-printed capsules after grayscale transformation, with image sizes of (A) 25 pixels, (B), 50 pixels, (C) 125 pixels, (D) 256 pixels, and (E) 512 pixels, Figure S4: MLTs performance for classifying SLA-printed tablets after grayscale transformation, with image sizes of (A) 25 pixels, (B), 50 pixels, (C) 125 pixels, (D) 256 pixels, and (E) 512 pixels, Figure S5: MLTs performance for classifying SLA-printed films after grayscale transformation, with image sizes of (A) 25 pixels, (B), 50 pixels, (C) 125 pixels, (D) 256 pixels, and (E) 512 pixels.
