# Supplementary material for: Virtually Possible: Enhancing Quality Control of 3D-Printed Medicines with Machine Vision Trained on Photorealistic Images
Source: Pharmaceutics. 2023 Nov 16;15(11):2630. doi: 10.3390/pharmaceutics15112630 (PMC10674815; doi:10.3390/pharmaceutics15112630)
Supplement: Supplementary file 1 [file pharmaceutics-15-02630-s001.zip › pharmaceutics-2645460-supplementary.pdf]

# Virtually Possible: Enhancing Quality Control of 3D-Printed Medicines with Machine Vision Trained on Photorealistic Images

**Siyuan Sun [1], Manal E. Alkahtani [1,2], Simon Gaisford [1], Abdul W. Basit [1], Moe Elbadawi [1,3,*] and Mine Orlu [1,*]**

[1] UCL School of Pharmacy, University College London, 29-39 Brunswick Square, London WC1N 1AX, UK
[2] Department of Pharmaceutics, College of Pharmacy, Prince Sattam bin Abdulaziz University, Alkharj 11942, Saudi Arabia
[3] School of Biological and Behavioural Sciences, Queen Mary University of London, Mile End Road, London E1 4DQ, UK
* Correspondence: m.elbadawi@qmul.ac.uk (M.E.); m.orlu@ucl.ac.uk (M.O.)

*Table S1. MLTs and their parameters for grid search.*

| MLT | Parameters |
|---|---|
| **Decision Tree** | Splitter = ['best', 'random'] |
| | max_depth = [None, 4,5,6,7,8] |
| | criterion = ['gini', 'entropy', 'log_loss'] |
| | min_samples_leaf = [1,2,3,4,5] |
| | max_features = ['auto', 'sqrt', 'log2'] |
| **Random Forest** | n_estimators = [2,3,4,5,6,7,8,9,10] |
| | max_features = ['auto', 'sqrt', 'log2'] |
| | max_depth = [4,5,6,7,8] |
| | criterion = ['gini', 'entropy'] |
| **Gradient Boost** | max_features = ['auto', 'sqrt', 'log2'] |
| | loss = ['log_loss', 'deviance', 'exponential'] |
| | learning_rate_init = [0.1] |
| | n_estimators = [2,3,4,5,6,7,8,9,10] |
| | max_depth = [4,5,6,7,8] |
| **Logistic Regression** | C = [1,2,3,4,5,6,7,8,9,10] |
| | penalty = [None, 'l1', 'l2', 'elasticnet'] |
| | solver = ['lbfgs', 'liblinear', 'newton-cg', 'newton-cholesky', 'sag', 'saga'] |
| **Multi-layer perceptron** | neurons = [1,10,25,50,100] |
| | activation = ['identity', 'logistic', 'tanh', 'relu'] |
| | learning_rate_init = [0.1, 0.001, 0.00001] |
| **k-nearest neighbour** | n_neighbors = [1,2,3,4,5,6,7,8,9,10, 12, 15] |
| | weights = ['uniform', 'distance'] |
| | algorithm = ['auto', 'ball_tree', 'kd_tree', 'brute'] |
| **Support Vector Machines** | C = [1,2,3,4,5,6,7,8,9,10] |
| | kernel = ['rbf', 'linear', 'poly', 'sigmoid'] |
| | shrinking = [True, False] |

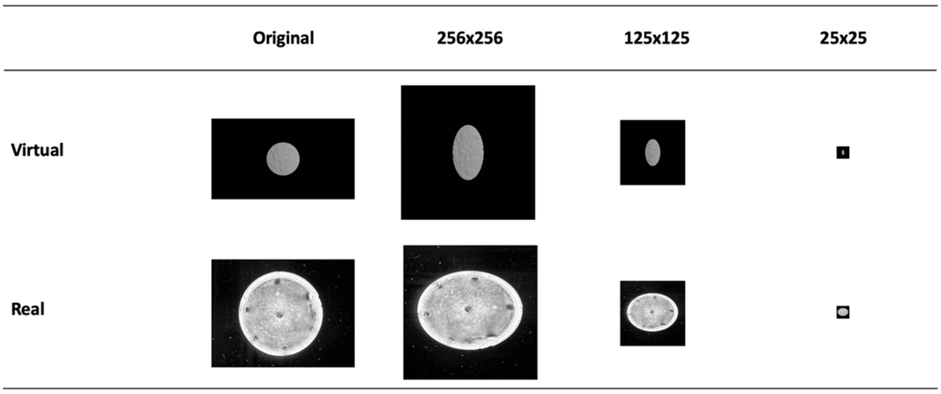


*Figure S1. Representative images of tablets following re-sizing.*

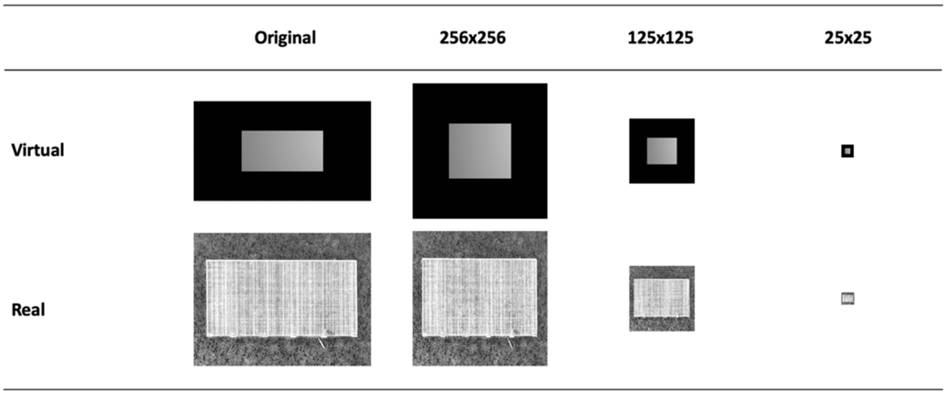


*Figure S2. Representative images of films following re-sizing.*

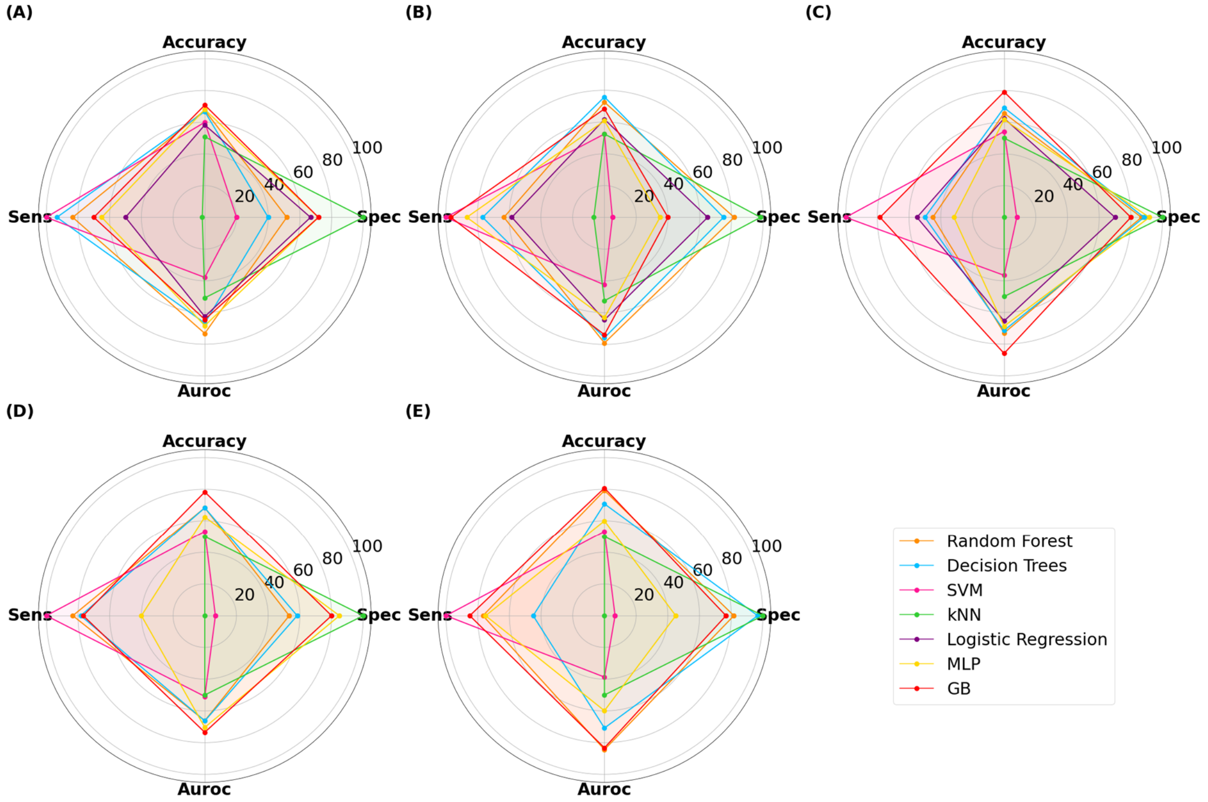


*Figure S3. MLTs performance for classifying SLA-printed capsules after grayscale transformation, with image sizes of (A) 25 pixels, (B), 50 pixels, (C) 125 pixels, (D) 256 pixels and (E) 512 pixels.*

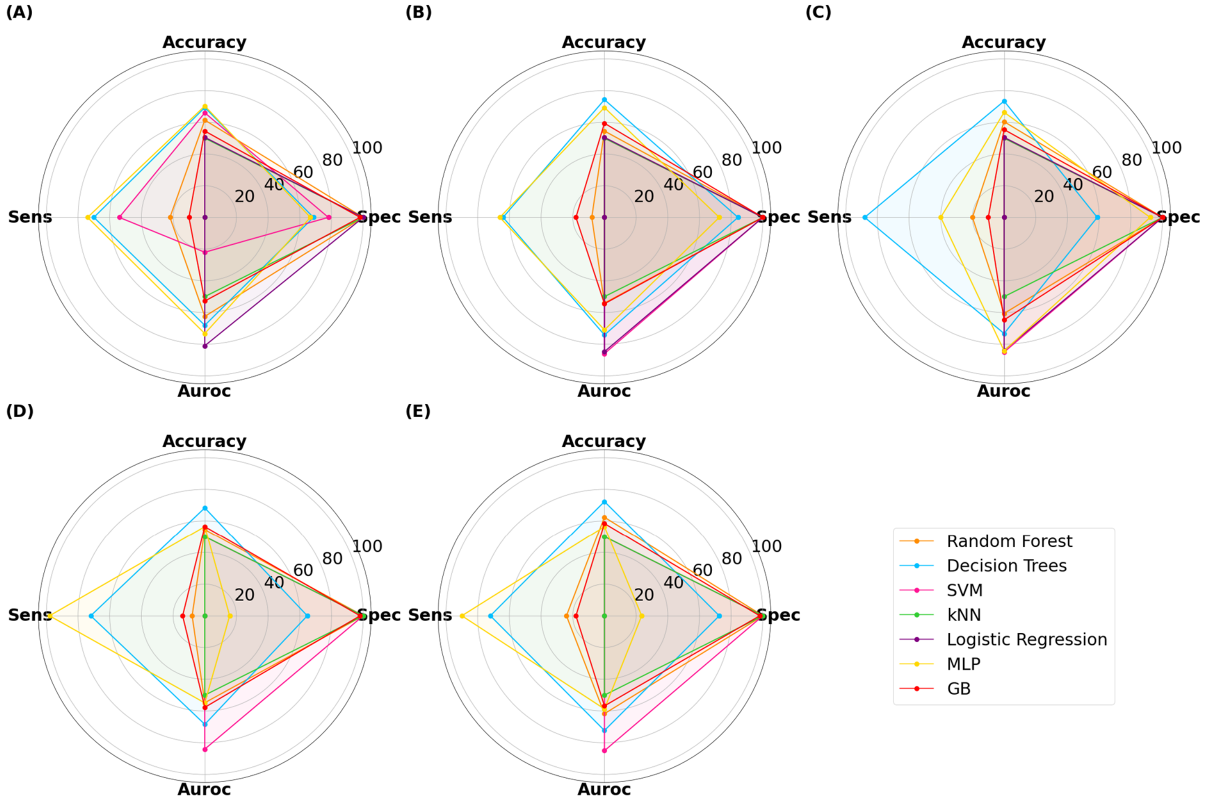


*Figure S4. MLTs performance for classifying SLA-printed tablets after grayscale transformation, with image sizes of (A) 25 pixels, (B), 50 pixels, (C) 125 pixels, (D) 256 pixels and (E) 512 pixels.*

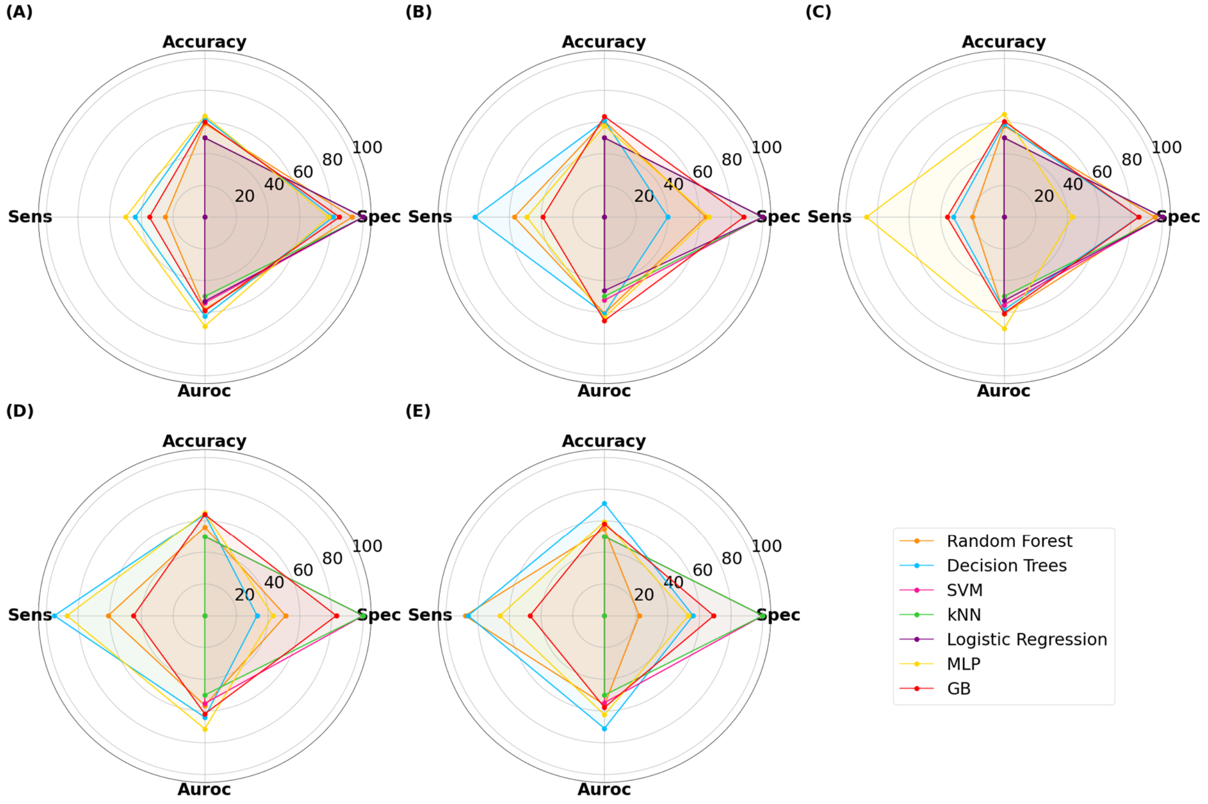

*Figure S5. MLTs performance for classifying SLA-printed films after grayscale transformation, with image sizes of (A) 25 pixels, (B), 50 pixels, (C) 125 pixels, (D) 256 pixels and (E) 512 pixels.*
